# *RAS* Mutation in Mucinous Carcinoma of the Ovary

**DOI:** 10.31557/APJCP.2019.20.4.1127

**Published:** 2019

**Authors:** Pinyada Panyavaranant, Chinachote Teerapakpinyo, Natkrita Pohthipornthawat, Shina Oranratanaphan, Shanop Shuangshoti, Surang Triratanachat

**Affiliations:** 1 *Division of Gynecologic Oncology, *; 3 *Division of Cyto-pathology, Department of Obstetrics and Gynecology, Faculty of Medicine, Chulalongkorn University and King Chulalongkorn Memorial Hospital,*; 2 *Chulalongkorn GenePRO Center, Research Affairs, Faculty of Medicine, Chulalongkorn University, Bangkok, Thailand. *

**Keywords:** Genetic mutation, mucinous, ovarian cancer, KRAS, survival

## Abstract

**Objective:**

This study was designed to identify genetic mutation in mucinous carcinoma of the ovary of the patients in King Chulalongkorn Memorial hospital, Bangkok, Thailand and study the relationship between genetic mutation and patients’ prognosis.

**Methods:**

Fifty cases of primary mucinous carcinoma of the ovary were selected. DNA was analyzed for genetic mutation using ColoCarta Panel v1.0 and MassArray^®^ System. Demographic data and clinical information of the participants were reviewed from electronic medical records and government data services.

**Results:**

Median of disease-free survival is 171.33 +/- 9.04 months and the median overall survival is 171.37 +/- 9.03 months. Twelve percent of the participants had recurrence and all of recurrent cases died from disease or its complication. We found three mutations which were *KRAS* (27 cases, 54%), PIK3CA (4 cases, 8%) and BRAF (1 case, 2%). Among the *KRAS*-mutated patients, the majority of the cases (25 cases, 92.6%) were in stage I. Recurrence and disease related mortality were not observed in the *KRAS* mutated patients.

**Conclusion:**

The genetic mutation analysis found three mutations which were *KRAS* 27 cases (54%), PIK3CA 4 cases (8%) and BRAF 1 case (2%) The ovarian mucinous carcinoma patients with *KRAS* mutation in our study showed excellent prognosis.

## Introduction

Ovarian cancer is approximately 3.6% of all cancers that occur in the female population.(Ferlay et al., 2015) Data from the United States found that 5% of mortality rate in female population results from ovarian cancer.(Atguden et al., 2016) In Thailand, ovarian cancer is the second most common cancer in female reproductive system. The incidence of new case is 6.2 cases of ovarian cancer per 100,000 population per year (Khuhaprema et al., 2013). Unfortunately, the screening tests such as pelvic examination, tumor markers or ultrasound are not effective in detecting early stage ovarian cancer (Abdalla et al., 2016). Therefore most ovarian cancers detected by doctors are in an advanced stage where treatment is more difficult and prognosis is poor. The origin of ovarian cancer cell cannot be clearly identified. However, a number of studies confirmed that gene mutations are a major cause of development of ovarian cancer. Therefore, studies on genetic mutation may play a role in the early detection of ovarian cancer or risk stratification management (Gentry-Maharaj et al., 2015). 

Epithelial ovarian cancer comprises of 90% of malignant ovarian neoplasms (Morgan et al., 2016). Epithelial ovarian carcinomas are primarily classified by cell type into serous, mucinous, endometrioid, clear cell, and Brenner (transitional) tumors corresponding to different types of epithelium in the ovaries (Shih Ie and Kurman, 2004). Studies on the genetic abnormalities of serous ovarian cancer are widely available. In Western countries, serous ovarian cancer is much more common than other cell types. Genetic knowledge leads to the development of targeted therapy in serous ovarian cancer. On the other hand, mucinous carcinoma which is rarely found in western countries (2-4% of all ovarian cancer) (Gurung et al., 2013), are commonly found in Thailand (11-24% of all ovarian cancer) and Asian countries (Khuhaprema et al., 2013). The genetic mutation of mucinous ovarian cancer has not been widely studied. The discovery of genetic abnormalities will be the basis for further development of mucinous ovarian cancer screening and specific treatment. Mucinous ovarian cancer is a mucin-producing tumor similar to tumors in others organs such as intestine, breast and lung. The common genetic mechanisms among the mucin-producing tumor development and growth is called RAS/RAF/MAPK, PI3K/AKT pathway (Hugen et al., 2015). Thus, it is assumed that the mutation in this group may share some abnormality of oncogenes. In our routine genetic testing for mucinous intestinal cancer, ColoCarta Panel v1.0 and MassArray^®^ are used in most patients. Both assays have the ability to identify 32 mutations in 6 oncogenes. Therefore, they were chosen to detect genetic mutation in mucinous ovarian cancer in this study.

As far as our knowledge, there are no studies about genetic mutation in ovarian mucinous carcinoma in Thailand. Moreover, there are no studies to evaluate association between the genetic mutation and prognosis including survival of mucinous ovarian cancer. Therefore, this study was designed to identify genetic mutation in mucinous carcinoma of the ovary and also study the relationship between genetic mutation and disease-free survival as well as overall survival of the patients.

## Materials and Methods

After approval process from the Institutional Review Board of King Chulalongkorn University was obtained, all the cases of mucinous carcinoma of the ovary that underwent surgery from 1999 to 2009 were reviewed. The information was retrieved from the tumor registry data of Gynecologic Oncology Unit, Obstetrics and Gynecology Department, Faculty of Medicine, Chulalongkorn University, Bangkok, Thailand. Cases were selected with the inclusion criteria of 18-80 years of age, complete formalin-fixed-paraffin-embedded (FFPE) blocks and medical records that confirmed mucinous carcinoma of the ovary. For confirmation that the mucinous carcinoma was primary from the ovary, all of the FFPE blocks were reviewed and the tumor area was selected by a gynecologic pathologist. The mucinous carcinomas of ovarian tissues were tested for key markers of the primary mucinous carcinoma of the ovary by immunohistochemistry staining processes. The key markers are: positive for CK7, negative for CK20 and CDX2. The non- gynecologic primary mucinous carcinomas were excluded from our study.

General characteristics and clinical information of the participants were reviewed from electronic medical records. Extracted data included the age at diagnosis, BMI, parity, presenting symptoms, family history of cancer, the stage of the disease, primary treatment and adjuvant of treatment. The selected cases were interviewed by mail or phone call about their recurrence history. Death, cause of death, disease-free survival and overall survival were collected from medical records and government data services. 

Recurrence was defined as the return of cancer after complete treatment and after a period of time when the cancer could not be detected (American Cancer Society, 2017). Disease-free survival was defined as the length of time after primary treatment for a cancer ends that the patient survives without any signs or symptoms of that cancer (National Cancer Institute, 2017). Death of disease was defined as the death event caused by ovarian cancer, complication of disease or treatment, excluding other causes of death. Overall survival was defined as the length of time from the end of primary treatment to the death of disease (National Cancer Institute, 2017).

Genetic testing is a multistep approach process. First, Hematoxylin and Eosin (H and E) stained slides from FFPE tumor samples were reviewed. The tumor area was selected for DNA extraction by a pathologist. The DNA samples were extracted using QIAamp^®^ DNA FFPE Tissue kit according to the manufacturer’s instructions (Qiagen). DNA concentration was measured at OD260 and DNA quality was determined by the ratio of OD260/OD280 using NanoDrop 2000c spectrophotometry (Thermo Scientific). Mutations were analyzed using ColoCarta Panel v1.0 and MassArray^®^ System following the manufacturer’s instruction (Agena Bioscience). The ColoCarta Panel v1.0 allows identification of 32 mutations in 6 oncogenes including *BRAF (D594V, V600E, V600K, V600L, V600R), HRAS (Q61L), KRAS (A59T, G12A, G12C, G12D, G12F, G12R, G12S, G12V, G13D, G61H, Q61L), MET (R970C, T992I), NRAS (G12C, G12V, G13C, G13V, Q61E, Q61H)*, and *PIK3CA (C420R, E542K, E545K, H701P, H1047R, Q546K, R88Q)*. In brief, 20 ng of DNA samples were amplified using sets of ColoCarta Panel v1.0 PCR primers following the cycling conditions: 95 °C for 2 min, 45 cycles of 95 °C for 30 sec, 56 °C for 30 sec, and 72 °C for 30 sec, and a final step at 72 °C for 5 min. Then, the amplified DNA was purified using shrimp alkaline phosphatase (Yildiz et al., 2012) at 37 °C for 40 min, and a final step at 85°C for 5 min. The primers extension was performed using iPLEX® Pro reaction cocktail and ColoCarta v1.0 extended primers (Agena Bioscience) and the primers extension reaction as follows: 95 °C for 30 sec, 40 cycles of 95 °C for 5 sec, and 5 cycles of 52 °C for 5 sec and 80 °C for 5 sec, and then a final step at 72 °C for 3 min. After, a cation exchange resin was used to remove salts, the products were spotted on 24-well SpectroCHIP array (Agena Bioscience). An HPLC-purified water and human genomic DNA were selected as the blank and wild type DNA control in each experiment. Mutation analysis was performed using the software MassArray Typer 4.0 (Agena Bioscience) using a cut off mutation frequency of 5.0% integrated with medium or high credibility.

After the genetic testing process, all of the data were recorded and analyzed by SPSS version 17. Demographic data were analyzed by descriptive method. Relationships among the stage of the disease, recurrence and death were analyzed by Pearson Chi-Square. Disease-free survival and overall survival were analyzed by Kaplan-Mayer method. Statistical significance was defined as p-value< 0.05

## Results

**Figure 1:**
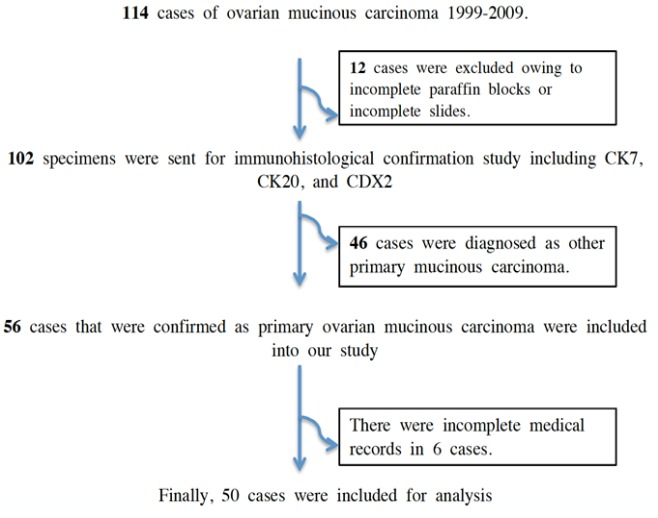
All of 114 Cases of Ovarian Mucinous Carcinoma Who were Diagnosed and Underwent Surgery from 1999 to 2009 Were Recruited. Finally, 50 cases were included into this study

All of 114 cases of ovarian mucinous carcinoma who were diagnosed and underwent surgery at King Chulalongkorn Memorial Hospital, Bangkok, Thailand from 1999 to 2009 were recruited (Figure 1). Twelve cases were excluded owing to incomplete FFPE blocks or slides. The remaining 102 specimens were tested for CK7, CK20 and CDX2. Forty six cases of non-gynecologic primary mucinous carcinoma were excluded. The remaining 56 cases that were confirmed as primary ovarian mucinous carcinoma were included in our study. Six cases were then excluded because of incomplete medical records. The remaining 50 cases were analyzed (Table 1).

**Table 1:** Demographic Data and Clinical Characteristics of the 50 Cases in This Study

Characteristic	
Mean Age +/- SD (Years)	46.06 +/- 13.81
Age (Range) (Years)	20-77
Parity (Cases)	
Nullipara	28 (56%)
Multiparous	22 (44%)
Symptoms † (Cases)	
Symptomatic	46 (92%)‡
Asymptomatic	4 (8%)
Family History of Cancer (Cases)	
No family history of cancer	45 (90%)
With family history of cancer	5 (10%)
Staging of ovarian cancer§ (Cases)	
I	37 (74%)
II	2 (4%)
III	6 (12%)
IV	5 (10%)
Recurrence of disease (Cases)	
Yes	6 (12%)
No	44 (88%)
Median disease-free survival +/- SD (Months)	171.33 +/- 9.04
Death of disease (Cases)	
Yes	6 (12%)
No	44 (88%)
Median overall survival +/- SD (Months)	171.37 +/- 9.03

†, Symptoms included abdominal mass, abdominal distension, abdominal pain and abnormal vaginal bleeding; ‡, In 46 symptomatic patients, 40 cases had only one symptom and 6 cases had two symptoms; §, According to International Federation of Gynecology and Obstetrics 2008.

**Table 2:** The Genetic Mutation Analysis Found Three Mutations which were KRAS, PIK3CA and BRAF

Genetic mutations	KRAS	PIK3CA	BRAF	HRAS	MET	NRAS
Number of cases	27 (54%)	4 (8%)	1 (2%)	0 (0%)	0 (0%)	0 (0%)

**Table3:** KRAS Mutation and Stage of Mucinous Ovarian Cancer. The majority of patients with mutated KRAS (92.6%) were in stage I

	KRAS Mutated (Cases)	KRAS Wild type (Cases)
Stage I	25 (92.6%)	12 (52.5%)
Stage II	0 (0%)	2 (8.7%)
Stage III	1 (3.7%)	5 (21.7%)
Stage IV	1 (3.7%)	4 (17.4%)
Total	27 (100%)	23 (100%)

Pearson Chi-Square (P-value = 0.013)

**Figure 2:**
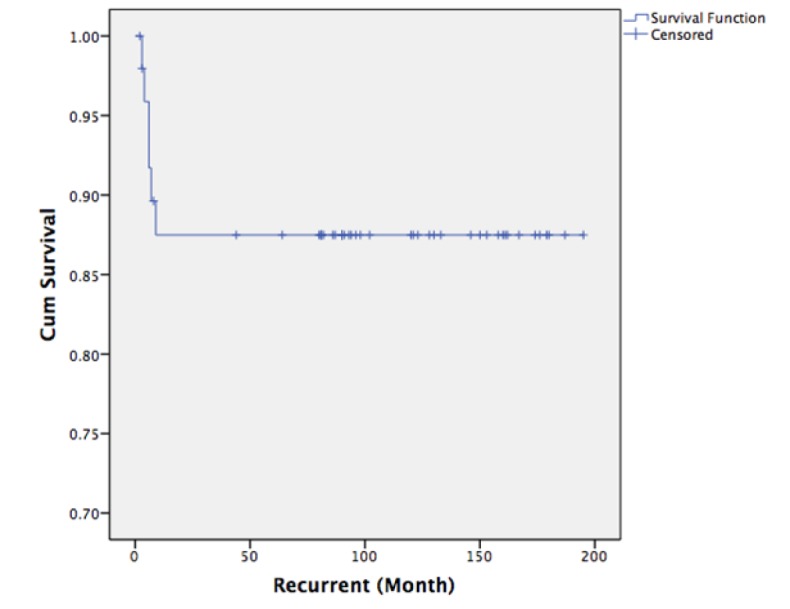
Kaplan-Meier Analysis of Disease-Free Survival: The Disease-Free Survival was Approximately 9 Months after the Last Treatment

**Figure 3:**
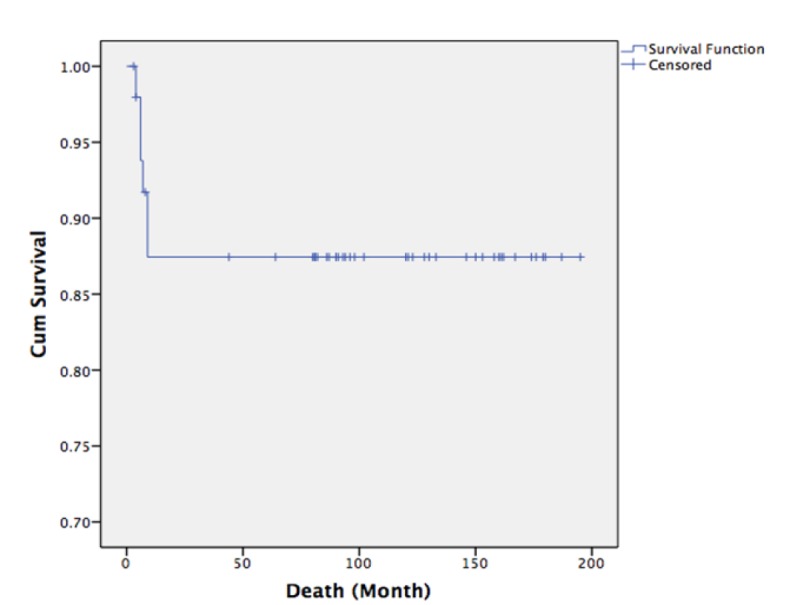
Kaplan-Meier Analysis of Overall Survival: All of the Recurrent Cases Were Dead from Disease or Its Complication in a Very Short Period of Time

**Figure 4:**
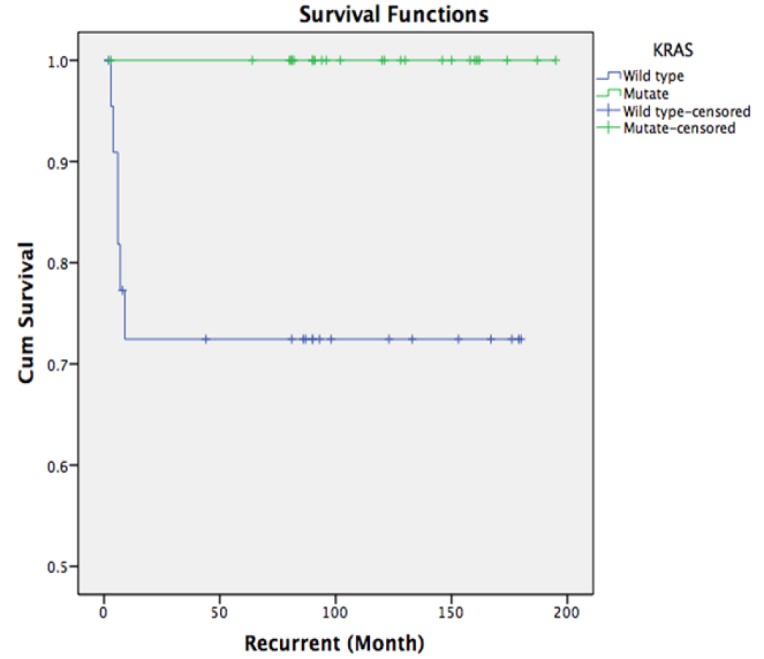
Kaplan-Meier Analysis of Disease-Free Survival in Wild-Type and KRAS Mutated patients: There was no recurrence and no disease related mortality in the KRAS mutated mucinous ovarian cancer patients

All recurrent cases had short disease-free survival. The disease-free survival was approximately 9 months after the last treatment. (Figure 2: Disease-free survival) All of the recurrent cases were dead from disease or its complication in a very short period of time. (Figure 3: Overall survival).

The genetic mutation analysis by ColoCarta Panel v1.0 and MassArray^®^ to identify 6 common oncogenes including *BRAF, HRAS, KRAS, MET, NRAS* and *PIK3CA*, found three mutations which were *KRAS 27* cases (54%), PIK3CA 4 cases (8%) and BRAF 1 case (2%) (Table 2).

To study the relationships between genetic mutations and other factors, we found relationship between the mutated *KRAS* and the stage of the disease. Twenty five patients with mutated *KRAS* (92.6%) were in stage I (Table 3). Moreover, BMI, parity, patient’s symptoms, family history of cancer were not significantly related to genetic mutation. 

The *KRAS* mutation was the most commonly found mutation in mucinous ovarian cancer in this study. All four cases that had the *PIK3CA* mutation were in stage IA of ovarian cancer and all of them also had the *KRAS* mutation. Only one case that had the BRAF mutation was in stage IIIc ovarian cancer.

To study the association between recurrence and the KRAS mutation, we found that there was no recurrence and no disease related mortality in the *KRAS* mutated mucinous ovarian cancer patients. The genetic study of the recurrent cases show that all of them had wild-type *KRAS*, p-value=0.004 (Figure 4)

## Discussion

It is now clearly known that different types of ovarian cancer differ not only in pathological characteristics, but also in the pathogenesis of cancer, including molecular activity and genetic abnormalities (Lim and Oliva, 2013; Teer et al., 2017). Many researchers trend to focus on the ovarian cancer development pathogenesis involving genetic defects and molecular dysfunction. In mucinous carcinoma of the ovary, previous studies clearly showed that the *KRAS* mutation was the important key point of the pathogenesis of the cancer (Lee et al., 2016; Teer et al., 2017). It is well known that hypothesis of a stepwise progression through the mucinous adenoma- mucinous borderline tumor- mucinous carcinoma sequence has been widely studied. Even the latest study by Lee et al., (2016) has also confirmed for this hypothesis. Interestingly, no previous research studied the relationship between genetic mutation and the prognosis of mucinous ovarian cancer.

In our research, we found three kinds of genetic mutation in mucinous carcinoma of the ovary which were *KRAS*, *PIK3CA*, and *BRAF*. The most common type was the *KRAS* mutation (54%) that was similar to earlier studies; 50% in Garrett’s (2001) study, 63% in Vereczkey’s (2011) study ,75% in Gurung’s (2013) study and 68.3% in Ryland’s (2015) study. A recent study from Taiwan also confirmed the *KRAS* mutation as an important factor in the ovarian mucinous adenoma-borderline tumor-carcinoma sequence (Lee et al., 2016). In cancer theory and the process of cell division, there are many genes that control the amplification signal. One of the important genes is the* RAS* family, which consist of *KRAS, HRAS *and *NRAS* (Li et al., 2015). The *KRAS* gene is a kind of oncogene. In the case of a *KRAS* gene mutation, abnormal intracellular signaling is turned on and the infinite uncontrolled cell proliferation is automatically manifested. When we compared the mutation of ovarian cancer to other mucin-producing tumors, they all have the same mutant genes which were *KRAS* and *BRAF*, but different in the percentage of mutation. For example in colon cancer, the *KRAS* mutation was found 22-39% and the BRAF mutation was found 2.8-6.6% (Li et al., 2015; Ye et al., 2015) While, the *KRAS* and *BRAF* mutations in mucinous ovarian cancer were 50-75% (Garrett et al., 2001; Gurung et al., 2013; Ryland et al., 2015; Vereczkey et al., 2011) and 3.5% (Perren, 2016) respectively. Nevertheless, the ovarian mucinous carcinoma patients with the *KRAS* mutation in our study showed excellent prognosis. Those patients have not had recurrence of the disease and still survive. There was a hypothesis about *RAS* mutational activation signaling pathway in ovarian cancer since 2003. This mechanism provided almost the case to indolent ovarian cancer type, very rare for metastatic type (Gemignani et al., 2003). In contrast to other cancers, the *KRAS* mutation of mucinous lung cancer or intestinal cancer encountered poor prognosis (Jun et al., 2016; Kadota et al., 2016). The relationship between the PIK3CA and mucinous ovarian cancer has not been clearly studied. In our study, there were only 4 cases of the PIK3CA mutation. The number of cases is too small to find any correlation with any factors. 

Using the Panel to detect genetic mutation with The ColoCartar Panel v1.0, *KRAS* mutation can be detected at *A59T, G12A, G12C, G12D, G12F, G12R, G12S, G12V, G13D, G61H, Q61L. *These all mutations of mucinous carcinoma of the ovary are reported in the COSMIC database (http://cancer.sanger.ac.uk/cosmic). However, future mutations may be found in other codons that are not currently reported in the COSMIC catalog of somatic mutations in cancer. As we know that any genetic testing tools can have false-positive results. For confirmation, all the 27 *KRAS* mutated samples were confirmed with a second technique that was pyrosequencing. In 27 *KRAS* mutated samples, the pyrosequencing showed a consistent result.

Our *KRAS* mutated patients had better prognosis and lower recurrence while all of the recurrent cases in our study had wild-type *KRAS*. Therefore, gynecologists should pay attention to the wild-type *KRAS* patients because of a chance of recurrence and poorer prognosis. 

Our study has limitations including; In each specimen which entered the study, the pathological cancerous lesions were selected for DNA extraction. The normal area of the same case was not compared. Secondly, this is an observational descriptive study which may have some recall bias about their family history of cancer and our test kits can not be able to detect other genetic mutations such as the *HER2* mutation. Currently, *HER2* amplification is found in about one fifth of mucinous ovarian cancer (Chang et al., 2016; Perren, 2016). 

This study provides further information about the genetic mutations of mucinous ovarian cancer which could be the basis for further development of mucinous ovarian cancer screening and specific treatment. In the near future, the involving genes, definite patient characteristic and environmental factors will be discovered and brought to the new line of care and management, especially targeted therapy. Although there is in vitro evidence that targeted therapy can be used for mucinous ovarian cancer, there are only a few studies on animal or human and further investigation is required (Sato et al., 2012). Two other mutated genes, *PIK3CA* and *BRAF*, have been found in this study. These mutated genes might be important factors in the development of mucinous ovarian cancer and may provide opportunities for further research.
